# AGO104 is a RdDM effector of paramutation at the maize *b1* locus

**DOI:** 10.1371/journal.pone.0273695

**Published:** 2022-08-30

**Authors:** Juliette Aubert, Fanny Bellegarde, Omar Oltehua-Lopez, Olivier Leblanc, Mario A. Arteaga-Vazquez, Robert A. Martienssen, Daniel Grimanelli

**Affiliations:** 1 DIADE, University of Montpellier, CIRAD, IRD, Montpellier, France; 2 Universidad Veracruzana, INBIOTECA, Xalapa, Veracruz, Mexico; 3 Howard Hughes Medical Institute, Cold Spring Harbor Laboratory, Cold Spring Harbor, New York, New York, United States of America; University of Guelph, CANADA

## Abstract

Although paramutation has been well-studied at a few hallmark loci involved in anthocyanin biosynthesis in maize, the cellular and molecular mechanisms underlying the phenomenon remain largely unknown. Previously described actors of paramutation encode components of the RNA-directed DNA-methylation (RdDM) pathway that participate in the biogenesis of 24-nucleotide small interfering RNAs (24-nt siRNAs) and long non-coding RNAs. In this study, we uncover an ARGONAUTE (AGO) protein as an effector of the RdDM pathway that is in charge of guiding 24-nt siRNAs to their DNA target to create *de novo* DNA methylation. We combined immunoprecipitation, small RNA sequencing and reverse genetics to, first, validate AGO104 as a member of the RdDM effector complex and, then, investigate its role in paramutation. We found that AGO104 binds 24-nt siRNAs involved in RdDM, including those required for paramutation at the *b1* locus. We also show that the *ago104-5* mutation causes a partial reversion of the paramutation phenotype at the *b1* locus, revealed by intermediate pigmentation levels in stem tissues. Therefore, our results place AGO104 as a new member of the RdDM effector complex that plays a role in paramutation at the *b1* locus in maize.

## Introduction

Paramutation is defined as the meiotically and mitotically heritable change in expression resulting from the interaction between specific alleles [1–5]. This phenomenon has been observed at four loci in maize, all encoding a transcription factor mediating flavonoid biosynthesis: *red1* (*r1*), *plant color1* (*pl1)*, *pericarp color1* (*p1*) and *booster1* (*b1*). Paramutation at *b1* is one of the best characterized systems [6–8]. It involves the highly transcribed *BOOSTER-INTENSE* (*B-I*) allele causing dark pigmentation in most tissues and the *BOOSTER’* (*B’*) allele which lower expression results in light pigmentation. When *B-I* and *B’* are combined, *B’* induces the meiotically stable *trans*-silencing of *B-I* and this conversion is permanent. In addition, *B-I* converted alleles acquire *B’* paramutagenic capacity and therefore can trigger secondary paramutation events in the next generation. *B’* and *B-I* are genetically identical and are hence refered to as epialleles. High transcription and full paramutagenicity (*trans*-silencing) at the *B-I* epiallele require the presence of at least five tandem repeats of a 853-bp sequence (*b1TR)* located ~100 kb upstream of the transcription starting site [6, 7]. The *b1TRs* produce 24-nucleotide (nt) small interfering RNAs (siRNAs) through the RNA-directed DNA Methylation (RdDM) pathway [8]. Previous studies demonstrated that paramutation has an establishment phase in developing embryos, but the irreversible change from *B-I* to *B’* likely occurs during the vegetative phase, owing to increasing methylation in *b1TRs* up to levels found in *B’* [9–11]. There is evidence that the RdDM pathway is critical for both establishment and maintenance of paramutation in maize [1, 12–17].

**Fig 1:**
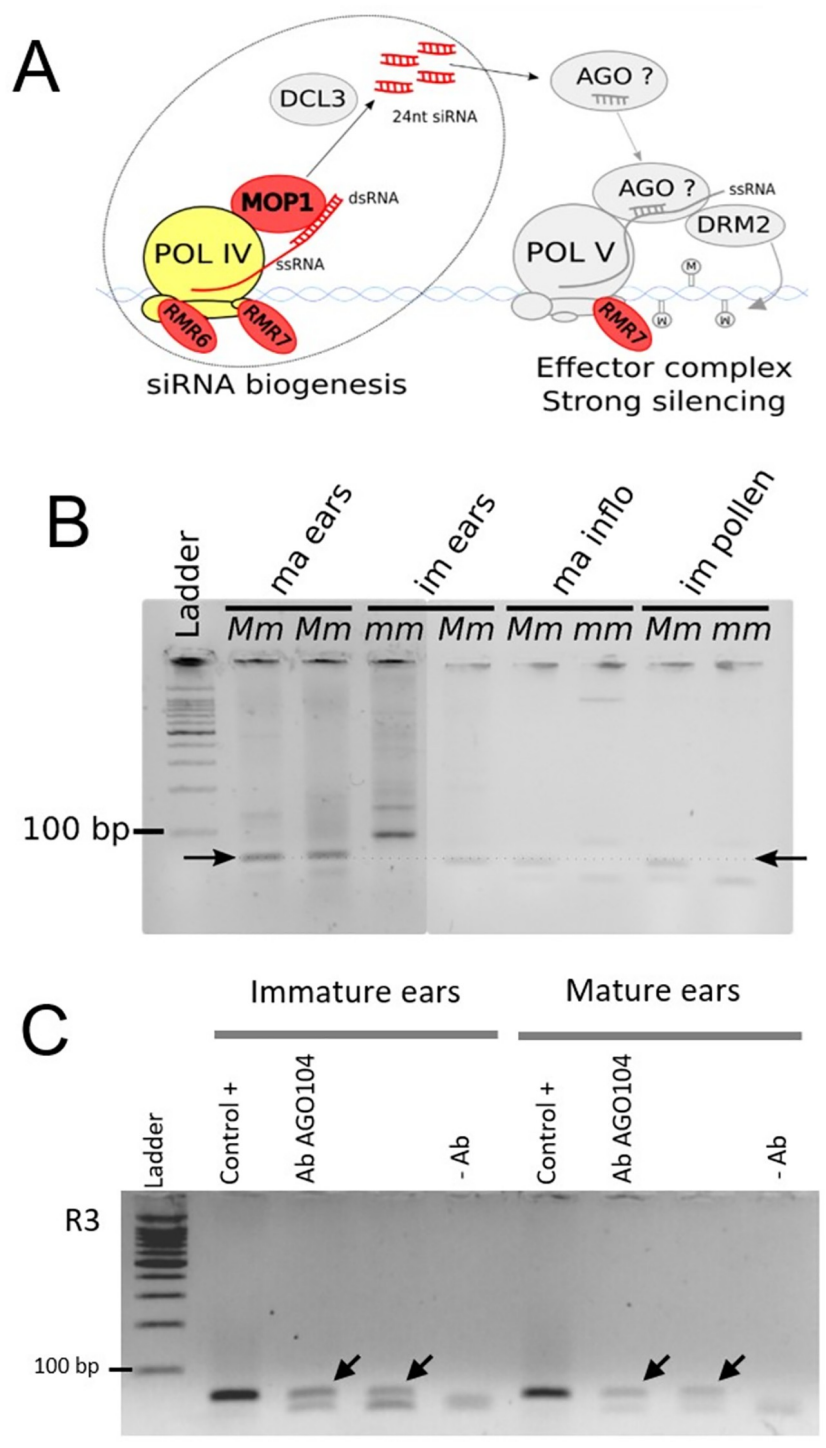
*b1TR* siRNAs and AGO104 interact in reproductive tissues. **A)** Identified (colored) and putative as based on homology with *A*. *thaliana* proteins (grey) RdDM members involved in small interfering RNAs biogenesis (left) and *de novo* DNA methylation (right) in maize. RdDM proteins involved in paramutation are shown in red. **B)** Stem-loop PCR for RdDM-dependent R3 siRNAs in immature (im) and mature (ma) reproductive tissues of *Mm* and *mm B’* plants. Arrows indicate the 67-bp expected band generated by R3. Ladder: Promega 100bp DNA Ladder Molecular Weight Marker. **C)** Stem-loop PCR of siRNAs extracted from IPs of AGO104 performed in tissues of *B’ Mop1/Mop1* plants. Control + are small RNAs extracted directly from reproductive tissues. AbAGO104 are small RNAs extracted from the IPs of AGO104. -Ab correspond to the mock immunoprecipitation samples (without Ab). Arrows indicate the 67-bp expected bands. Ladder: Promega 100bp DNA Ladder Molecular Weight Marker.

The RdDM pathway deploys two main functions, the biogenesis of 24-nt siRNAs and the use of these siRNAs for guiding sequence-specific DNA methylation and transcriptional repression (Fig 1A). In the first step, RNA POLYMERASE IV (POL IV) transcripts are immediately converted into double-stranded RNAs (dsRNAs) by MEDIATOR OF PARAMUTATION1 (MOP1), the homolog of RNA-DEPENDENT RNA POLYMERASE2 (RDR2) in *Arabidopsis thaliana*. DICER-LIKE3a (DCL3a) then slices these dsRNAs into 24-nt siRNAs [18, 19] which are necessary to the effector complex to induce DNA methylation at either CG, CHG or CHH sites (where H = A, T, or C) (Fig 1A). Few members of the effector complex were identified in maize, although they were extensively described in *A*. *thaliana* (reviewed in [20–23]). In *A*. *thaliana*, it initiates with *At*AGO4/6/9 [24, 25], that load and guide siRNAs to long non-coding scaffold transcripts generated by POL V [22, 26, 27]. This AGO-siRNA complex then recruits DOMAINS REARRANGED METHYLTRANSFERASE1 (DRM1) and DRM2 at specific target loci to mediate DNA methylation [20, 22, 23] (Fig 1A).

To date, RdDM members found to affect paramutation in maize include MOP1 [12, 28] and two REQUIRED TO MAINTAIN REPRESSION (RMR) proteins, RMR6/MOP3 that encodes the largest subunit of POL IV [13, 29, 30] and RMR7/MOP2 that encodes a subunit shared between POL IV and POL V [16, 31]. Both MOP1 and RMR6/MOP3, that act upstream dsRNAs biogenesis, are essential to maintain paramutation states at *b1*, as illustrated by the dark purple phenotypes resulting from the *mop1* and *mop3* recessive mutations [28]. The role of RMR7/MOP2 in paramutation remains unclear as the properties of *mop2* alleles vary, i. e. recessive for restoring *b1* repeats expression and dominant for disrupting paramutation, and the requirement of MOP2 for guiding DNA methylation remains speculative [30]. Therefore, no specific actor of the RdDM effector complex (later called effectors) has been identified yet in maize (Fig 1A).

*At*AGO4 regulates gene silencing through RdDM in *A*. *thaliana* [32, 33], and we first considered its two closest homologs in maize, *Zm*AGO105 and *Zm*AGO119 for our study. However, possible complementation resulting from high sequence similarity between the two maize sequences [34] renders arduous their functional characterization. On the other hand, *At*AGO9 is a close paralog of *At*AGO4, although they load different siRNAs [32]. *At*AGO9 also plays a crucial role in RdDM in *A*. *thaliana* whereby its expression in reproductive tissues is of particular interest for the establishment of paramutation. *Zm*AGO104 has been well characterised and was proposed as a putative homolog of *At*AGO9 in maize (hereafter referred to as AGO104) [34]. The goal of this work was to determine whether AGO104 is an effector of the RdDM complex and whether it is involved in paramutation. Using small RNA-immunoprecipitation of AGO104 combined with next-generation sequencing, we show that AGO104 binds RdDM-associated 24-nt siRNAs and that *b1TRs* of the *b1* enhancer region are among the RdDM target loci. Finally, we designed a reverse genetics approach using *mop1-1;ago104-5* stocks to validate functionally the role of AGO104 in paramutation. Taken together, this data indicate that AGO104 is a member of the RdDM effector complex in maize and that it participates in paramutation at the *b1* locus. This research provides a deeper understanding of the molecular mechanisms underlying paramutation as well as new insights into the role of RdDM in maize.

## Results

### AGO104 binds RdDM-associated siRNAs in reproductive tissues

To determine if AGO104 binds RdDM-associated siRNAs, we selected *B’ mop1-1/mop1-1* mutants (*mm*) that was introduced in the *B’* genetic background, and disrupts the RdDM pathway by decreasing the amounts of 24-nt siRNAs while it remains fully operational in heterozygous (*Mm*) plants [28, 35].

To validate our hypothesis that AGO104 is a functional homolog of *At*AGO9, we first extracted total small RNAs from immature (at sporogenesis) and mature (at gametogenesis) ears, and mature pollen from both *Mm* and *mm* plants (*B’*) and investigated by stem-loop PCR the expression of 24-nt siRNAs previously identified as “RDR2-sensitive” [35, 36]. The detection of one of these siRNAs, R3, in ears and pollen of *Mm* plants (*B’*), but not in *mm (B’)* reproductive tissues (Fig 1B) confirmed that R3 siRNAs biogenesis is RdDM-dependent. Previously published immunolocalization using a specific antibody directed against AGO104 showed that AGO104 is present in mature and immature ears [34], suggesting a possible co-expression of AGO104 and R3. This led us to perform AGO104 RNA immunoprecipitation (RNA-IP) in reproductive tissues from *Mm (B’)* plants followed by sRNAs extraction and stem-loop PCR for R3 amplification. Using Singh et al., (2011) antibody for AGO104 immunoprecipitation, we amplified a clear band of the expected size, indicating that AGO104 binds R3 siRNAs in maize reproductive tissues (Fig 1C). This strongly suggests that AGO104 acts in RdDM, downstream of siRNA biogenesis (hence, downstream of MOP1).

**Fig 2:**
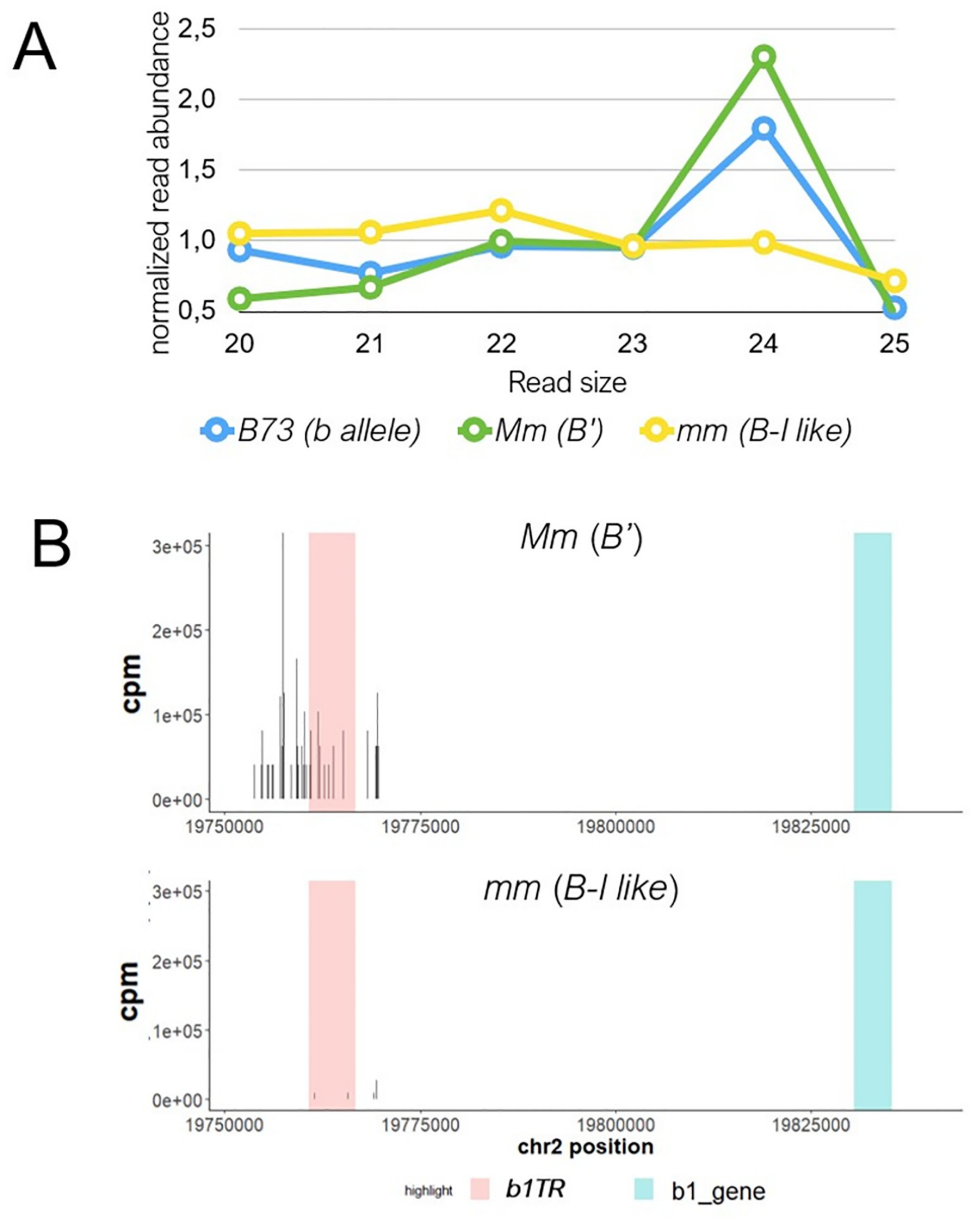
RNA-IP sequencing (RIP-seq) of AGO104-loaded small RNAs in mature ears of B73 (*b*), *Mm* (*B’*) and *mm* (*B-I like*) individuals. **A)** Size distribution of reads normalized to 1. **B)** Distribution of 24-nt reads within the 100-kb region that includes the *b1TR* (red box) and the *b1* gene (blue box). *x* axis shows the B73/*b1TR*s composite reference map used for aligning reads. Vertical black bars indicate normalized read counts (CPM: Counts Per Million).

Finally, we used Illumina sequencing of libraries prepared from the small RNAs previously extracted from AGO104 in immature ears of plants producing normal (B73 with the *b* allele and *Mm* with the *B’* epiallele) and reduced (*mm* with the *B’* epiallele) amounts of 24-nt siRNAs. About two million cleaned reads were generated from each library and aligned onto the B73 reference genome. Mapped read (20–25 nt) counts were normalized using the transcripts per million (TPM) method. All genotypes displayed a similar chromosome-scale coverage (S1 Fig). Note that we used this method aiming at normalizing all backgrounds to similar expression levels to compare global chromosome coverage rather than differences in siRNAs expression. Read size distribution in plants producing reduced amounts of siRNAs (*mm; B’*) revealed that AGO104 can bind 21 and 22-nt small RNAs (Fig 2A) whereas in plants with regular abundance of siRNAs (B73; *b* and *Mm; B’*) it binds preferentially 24-nt siRNAs. This change is probably caused by a 24-nt siRNAs decreased abundance in *mm* plants (*B’*) rather than by a change of AGO104 specificity [35]. However, these results indicate that AGO104 binds 24-nt siRNAs in a non-mutant background, which strengthens our conclusion that AGO104 is likely an effector of RdDM.

### AGO104 binds 24-nt siRNAs generated from b1TR sequences

As an effector of RdDM acting in reproductive tissues of maize, we wanted to determine whether AGO104 is a factor contributing to paramutation. It is worth noting that the *mop1-1* genetic stocks used in this research contain the *B’* epiallele (ie, its enhancer region harboring seven *b1TR* sequences). The *b1* repressed state of *B’* epiallele is impaired in *mm* plants, which causes a dark purple pigmentation, similar to that of plants carrying the *B-I* epiallele. However, the *mop1-1* mutation is recessive and the *B’* repressed state as well as light pigmentation are restored in progeny derived from crosses between *mm* and WT MOP1 plants [28]. In other words, *Mm* and MOP1 plants display the same lightly pigmented phenotype. Therefore, *B’* epialleles transmitted by *mm* plants are referred to as *B-I like* hereafter.

As a first step, we verified that AGO104 has the capacity to load 24-nt siRNAs associated with paramutation. To achieve this, we used the 20–25 nt normalized reads from the above *Mm (B’)* and *mm (B-I like)* libraries and mapped them onto a composite segment assembled using the 100-kb region of the B73 reference genome centered on the *b1* enhancer region which we replaced by the *b1TR* repeats found in the *B’* genetic background (GenBank accession AF483657) [6]. The *b1* enhancer region in B73 was identified using sequence homology with the *b1TR* repeats. Interestingly, 24-nt small RNAs extracted from AGO104 in the *mm* mutant (*B-I like*, reduced amounts of 24-nt siRNAs) failed to map to the *b1TRs*. However, 24-nt small RNAs extracted from AGO104 in *Mm* plants (B’, producing normal amounts of 24-nt siRNAs) mapped correctly to the *b1TRs* region (Fig 2B). This data indicates that AGO104 from *Mm* (*B’*) binds 24-nt siRNAs that are produced from the *b1TR*. Our results support well the conclusion that AGO104 is a strong candidate factor for paramutation.

### *ago104-5* mutation disrupts paramutation at the b1 locus

While *B’* paramutagenic epialleles are highly stable, naïve *B-I* paramutable epialleles are unstable and can spontaneously change into *B’* with a wide range of frequencies (from 0.1 up to > 50%) depending on the genetic background [9, 16]. To avoid such instability and to ensure that the anthocyanin biosynthesis pathway is functional in the genetic background used, we took advantage of the *mop1-1* mutation properties that do not alter the *B’* paramutated status but causes a *B-I* dark purple phenotype in homozygous (*mm)* plants, hereafter identified as *B-I like*. Both *B’* and *B-I like* phenotypes are predictable and easily trackable. Paramutagenicity and phenotypes of *mm* (*B-I like*) plants were extensively studied upon crossing with plants carrying neutral *b* alleles and with *Mm* (*B’*) plants and both progenies always resulted lightly pigmented *B’* [12, 28]. We rationalized that depleting AGO104 in this *B’* progeny would ensure the stability of a functional anthocyanin pathway and allow to determine the role of AGO104 in paramutation, i.e. does AGO104 loss of function allow the reversion of *B’* epialleles to *B-I like* or *B-I* epialleles?

**Fig 3:**
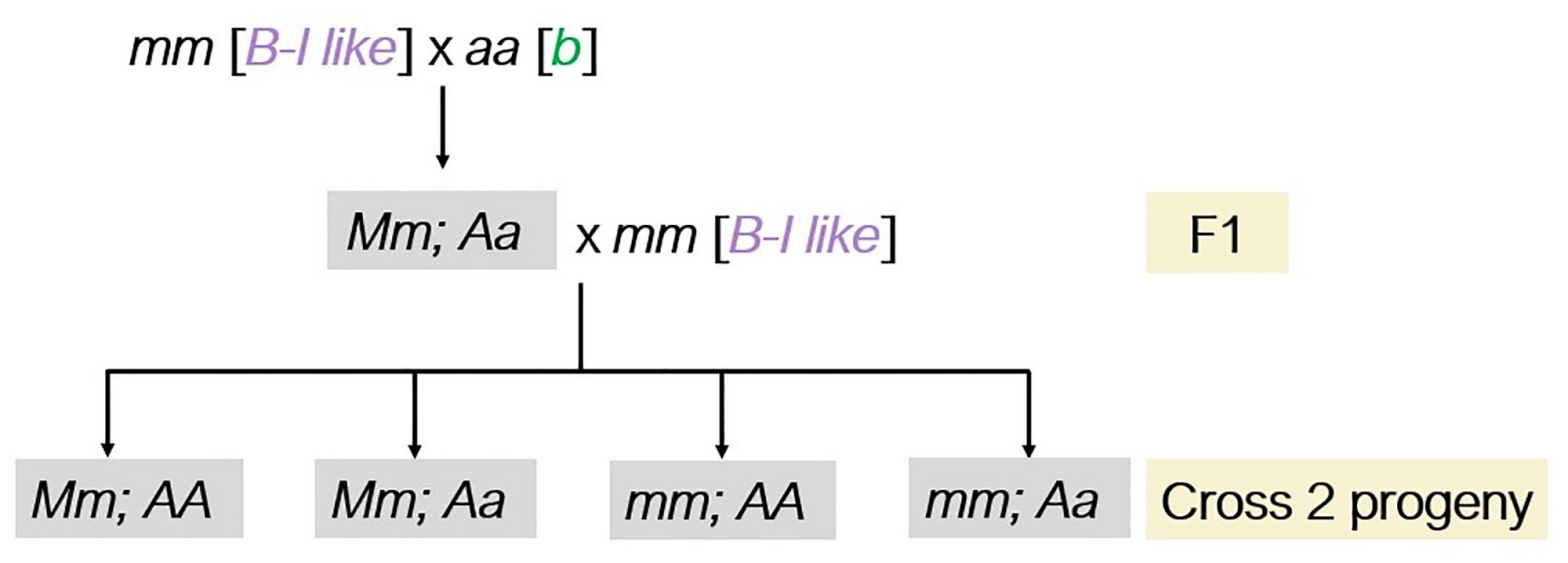
Crossing scheme used for a reverse genetic screen designed to investigate AGO104 contribution to paramutation. Alleles in genotype descriptions are as follows: *M*: *mop1; m*: *mop1-1; A*: *ago104; a*: *ago104-5; b*: neutral *b1* allele; *B-I like*: epiallele from a *mm* plant. Pigmentation phenotypes are indicated in squared brackets.

To achieve this, we selected *ago104-5*, a Mutator-induced allele previously characterized as a dominant knockout allele creating defects during female meiosis and apomixis-like phenotypes [34] and available in the B73 background that carries a neutral *b* allele [37]. To combine the *ago104-5* mutation and the *mop1-1* mutation and generate a reverse genetics population, we first crossed *mm* plants (dark purple, *B-I like*) with *ago104-5* (*aa*) plants (green, neutral *b*) (Fig 3). We then backcrossed F1s (*Mm;Aa*) to the *mm* mutant (Fig 3) and generated progenies either functional (*Mm*) or deficient (*mm*) for 24-nt siRNA biogenesis. Previous reports for crosses between *Mm* plants (*B’*) and B73 plants (same genetic background as *ago104-5*, *b* allele) indicate no significant effect on plant pigmentation [38, 39]. This strongly suggests that the B73 genome does not harbor factors affecting *B’* expression. As the *mop1-1* mutation is recessive and assuming that the ago104-5 mutation is dominant as shown by Singh et al (2011), the *Mm* individuals represent the population of interest for our study as we anticipate the recovery of plants with a functional RdDM pathway (*Mm;AA*) and plants defective for the RdDM effector complex (*Mm;Aa*). We genotyped all produced plants at both the *mop1* locus and the *ago104* locus and evaluated stem pigmentation at 46 and 56 days post-seeding (dps). Finally, to control environmental effects, we also evaluated stem pigmentation of both *Mm* (*B’)* and *mm* (*B-I like*) plants derived from stocks segregating the *mop1-1* allele only.

**Fig 4:**
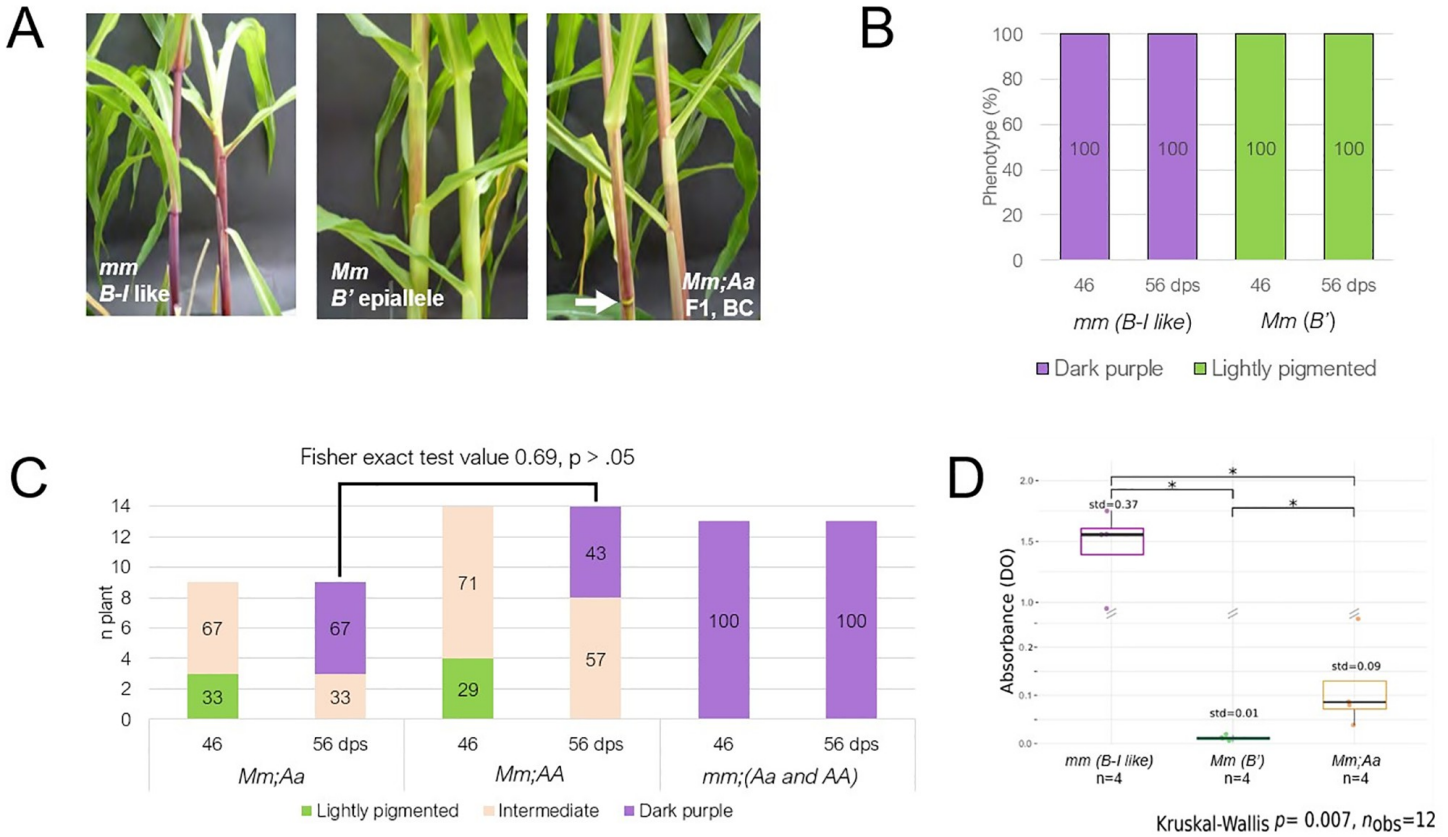
Pigmentation phenotypes observed at 46 and 56 days post seedling (dps). **A**) Pictures of stem phenotypes at 46 dps in (left-to-right): *mm* control plants (*B-I like*, dark purple pigmentation); *Mm* control plants (*B’*, light pigmentation); *Mm;Aa* cross 2 progenies (intermediate pigmentation). The white arrow indicates a typical unpigmented node. **B and C**) Pigmentation phenotypes in control plants (with n > 25 for each control) and in cross 2 progenies, respectively. Numbers in bars are percentages. **D)** Absorbance at 550 nm of anthocyanins extracted using 1 g of stem tissue from 56 dps plants. std is the standard deviation. y axis scale is shown at .05 intervals between 0 < DO < 0.25 and 0.25 intervals between 1 < DO < 2.

As expected from previous works using control plants (segregating the *mop1-1* allele only) [12, 28], all *mm* plants (*B-I like*, n>25) were dark purple at 46 and 56 days post seeding (dps), while all *Mm* plants (*B’*, n>25) were lightly pigmented (Fig 4A and 4B). Similarly, *mm* progeny from cross 2 (*B-I like*, n = 13) all displayed the same dark purple phenotype as seen in control *mm* plants and regardless of the *ago104* genotype (Fig 4C). Since MOP1 acts upstream of AGO104 in RdDM, this indicates that AGO104 unlikely contributes to paramutation through another, yet unknown, mechanism. Interestingly, both F1 plants and *Mm* progenies derived from cross 2 (n = 14 and n = 23, respectively) displayed a new phenotype with increasing levels of stem pigmentation between 46 and 56 dps, therefore suggesting gradual reversion of the *B’* allele to a paramutable state (Fig 4A). Interestingly, contrary to the canonical *B-I like* phenotype in *mm* plants, stem nodes typically lacked pigmentation (Fig 4A), indicating that *B’* paramutagenicity is likely maintained in this tissue. These observations are coherent with the dominance of the *ago104-5* mutation. Plants with this intermediate stem pigmentation midway between the typical *B’* and *B-I* stem pigmentations as well as a weak node pigmentation were classified as displaying “intermediate pigmentation”. Plants that displayed a dark purple stem and dark node pigmentation were classified as “dark purple”. Plants that displayed weak or no stem and node pigmentation were classified as “light purple”. Detailed analysis in *Mm* plants from cross 2 (n = 23, F1 plants were not formally evaluated) revealed that, at 46 dps, 30% (n = 7) of plants showed a typical light phenotype, while the remaining plants (n = 16) exhibited a partially reverted paramutation phenotype with intermediate levels of pigmentation and nodes lacking pigmentation (Fig 4A–4C). Pigmentation turned darker over time in all *Mm* progenies, none of which exhibiting at 56 dps the lightly pigmented stem typical of *B’* plants. At this stage, 52% of the progeny (n = 12) reached an intensity similar to that conferred by the *B-I like* epiallele in *mm* mutants, and the remaining plants (n = 11) reached intermediate pigmentation levels (Fig 4C).

### *ago104-5* mutation alters B’ paramutation when transmitted through meiosis

Our genetic screening revealed that the *Aa* genotypic condition causes a third phenotype marked by intermediate levels of pigmentation and different, at least visually, from the two hallmark phenotypes of paramutation at *b1* (see control plants in Fig 4B). To further this observation, we quantified anthocyanins by spectrophotometry in extracts obtained from stem tissues collected at 56 dps from plants with dark purple pigmentation (*mm*, *B-I like*), light pigmentation (*Mm*, *B’*), as well as intermediate levels of stem pigmentation (*Mm;Aa* progeny from cross 2). Both Kruskal-Wallis test (p = .007) and multiple pairwise comparison test (p = .029) indicated significant differences in pigment quantification among the three classes (Fig 4D). These findings validate the existence of three different *b1* paramutation phenotypes. It suggests that a gradual release of *B’* silencing allowed an increasing in anthocyanin production for all progenies, without however reaching the levels observed for *B-I like* in the *mop1-1* condition.

*Mm* progenies used here varied for the *Ago104* genotype, and *AA*:*Aa* followed the expected 1:1 ratio (Chi2 value 1.09, p < .05). The number of plants in each pigmentation group (dark purple, light purple and intermediate) between *Mm;Aa* and *Mm;AA* at 56 dps in the progeny of cross 2 do not significantly differ (Fisher exact test value 0.69, p > .05; see Fig 4 for category numbers). In other words, the intermediate phenotype identified in the *Mm* progeny from cross 2 happens with similar proportions in *Aa* and *AA* plants in the progeny of cross 2, which was not expected as *AA* plants do not carry the *ago104-5* mutation. This suggests that AGO104 deficiency either induces a parental effect on progeny pigmentation or heritably releases silencing of *B’* epialleles. Therefore, mutation of *ago104* alters the paramutation state of *B’* epialleles when transmitted through meiosis.

## Discussion

Based on sequence similarities previously reported [34], we argue that *ago104* in maize encodes a functional ortholog of *AtAGO9*, an assumption strongly supported by our analysis of small RNAs co-immunoprecipitated with AGO104. In particular, we show that AGO104 preferentially recruits 24-nt small RNAs, including those generated from *b1TR*s and involved in paramutation [25].

As previously shown, several “alternative” RdDM pathways enable the synthesis of 24-nt siRNAs without the involvement of RDR2/MOP1 in both *A*. *thaliana* [40] and maize [35, 41]. However, the production of 24-nt small RNAs in the *mop1-1* mutant is partially replaced by 22-nt small RNAs [35]. This supports our results by which AGO104 proteins in *mop1-1* mutant did not carry 24-nt siRNAs, and loaded preferentially 22-nt RNAs (Fig 2A). A possible explanation for this might be that the 22-nt small RNAs in *mop1-1* mutant contribute to rescue AGO104, but they do not mediate paramutation at the *b1* locus.

Our reverse genetic screening performed on *ago104-5* mutants broaden our understanding of AGO104 involvement in paramutation. Paramutation at the *b1* locus involves the *B-I* and *B’* epialleles, respectively associated with the typical intense and light plant pigmentation [42]. Here, our reverse genetics approach combining *ago104-5* and *mop1-1* mutations unveiled an intermediate pigmentation phenotype that turns darker over time (Fig 4C). However, although pigmentation in these plants seems to reach that of *mm* plants at 56 dps, quantification using spectrophotometry showed that *mm* plants produce higher levels of anthocyanins. Previous description of the *mop2* mutant also reported pigmentation changes over time that never reaches *mm* plants levels [16]. Both *mop2* and *mop1* mutants alter siRNAs production and potentially have effects beyond those resulting from RdDM downregulation (reviewed in [40]). In contrast, *ago104* mutations perturb RdDM targeting but not 24-nt siRNA production nor their possible contribution to paramutation through other regulatory mechanisms (reviewed in [32]).

Interestingly, all *Mm* plants (F1s and cross 2 progeny) displayed the same intermediate phenotype, demonstrating that AGO104 is an effector of paramutation and suggesting that the *ago104-5* mutation does not allow a complete reversion to the *B-I* dark purple phenotype. Other AGO proteins, such as AGO105 and AGO119, may complement AGO104 loss of function by restoring silencing at *b1* through *b1TR* siRNAs loading and, thus, preventing the full reversion to the *B-I* phenotype. Furthermore, both F1 plants and their *Mm;AA* progeny displayed an intermediate phenotype, suggesting that the *ago104-5* mutation alters the *B’* paramutation state through meiosis, and disrupts the heritability of paramutation at the *b1* locus. Such reversion of paramutation was previously described at the *Pl’* allele in the *mop1-1* mutant [28]. It is worth noting that the RMR7/MOP2 subunit of both POL IV and POL V is required for paramutation, although POL V is not involved in the phenomenon [16, 31]. Therefore, it is possible that AGO104 might be involved in paramutation independently of RdDM, through an yet-unknown pathway responsible for DNA methylation.

Consistent with our results of RNA-IP, previous studies have demonstrated that AGO104 is located exclusively in reproductive tissues (i.e. female and male meiocytes, egg cells, and embryos) [34], where paramutation is at least partly established [9–11]. Interestingly, *b1* is expressed in somatic tissues only [8, 37], where maintenance of paramutation takes place and where AGO104 is not expressed. Hence, AGO104 is probably involved in the establishment rather than the maintenance of paramutation. Interestingly, we observed green nodes in *Mm* progeny from cross 2 with intermediate levels of stem pigmentation. No previous research has been conducted to study the specific behaviour of meristematic tissues in paramutation, but the expression of developmental regulatory genes in maize is most often controlled by regulators of paramutation such as MOP1 and RMR6/MOP2 [28, 29, 43]. Therefore, it is possible that meristematic tissues possess backup mechanisms to regulate their development and, at the same time, can establish paramutation contrary to somatic tissues.

In this study, we demonstrated that AGO104 binds RdDM-associated 24-nt siRNAs in maize. We also confirmed that AGO104 binds paramutation-associated siRNAs by sequencing small RNAs loaded onto AGO104, and our reverse genetic approach validated the functional role of AGO104 in paramutation at the *b1* locus. AGO104 is involved in the establishment of paramutation in the reproductive tissues of maize, most likely through its function in the effector complex of the RdDM pathway. While other AGO proteins might play similar functions in RdDM and paramutation, our findings shed new light on the mechanisms mediating both the establishment and the transmission of paramutation in maize.

## Materials and methods

### Plant material

The B73 inbred line was provided by the Maize Genetics Cooperation Stock Center (University of Illinois, Urbana/Champaign, USA). The Trait Utility System for Corn (TUSC) at Pioneer Hi-Breed (Johnston, Illinois, USA) provided *ago104-5* stocks and V.L. Chandler (University of Arizona, Tucson, AZ, USA) provided the *mop1-1* mutant in the *B’* genetic background. Plants were grown in a greenhouse at the French National Research Institute for Sustainable Development in Montpellier, France, with 14 hours day light (26°C during the day, 20°C at night). For all these plants, inflorescences were partially dissected to evaluate pollen developmental stages with a Zeiss confocal microscope. We snap froze and stored at -80°C both inflorescences collected at sporogenesis and gametogenesis stages (respectively, immature and mature inflorescences), and pollen during sporogenesis (immature pollen). Ears at sporogenesis and at gametogenesis were selected based on their length (3 to 5 cm of length for immature ears and > 5 cm for mature ears) and the presence of silks, and were immediately snap frozen in liquid nitrogen and stored at -80°C.

### Genotyping

Total genomic DNA was extracted from seedling tissues using a standard CTAB procedure. After quality check for integrity and quality, DNA concentration was quantified using a NanoDrop spectrophotometer. Genotyping was performed by PCR using 20 μl reactions containing 200 ng DNA, 1 μL of 10 μM of forward and reverse primers (see S3 Table) and Quick-Load Taq 2X Master Mix (NewEngland Biolabs). For amplifications, sample preparations were denatured for 3 min at 95°C and subjected to 35 cycles as follows: 15 s at 95°C for denaturation, 15 s at 55°C for annealing and, 60 s and 165 s extension at 68°C for *mop1-1* and *ago104-5*, respectively. Amplification products were loaded in 1.5% agarose gels, electrophorized at 100 V for 20 min and visualized by ethidium bromide staining.

### Small-RNA immunoprecipitation

Protocols were adapted from [25] using two biological replicates per genotype. Tissues were grinded with liquid nitrogen and a Dounce homogenizer. Resulting powder was placed in a Falcon tube with 3 volumes of extraction buffer (20 mM Tris HCL pH 7.5, 5 mM MgCl_2_, 300 mM NaCl, 0.1% NP-40, 5 mM DTT, 1% protease inhibitor (Roche Tablet), 100 units/mL RNase-OUT (Invitrogen). Samples were vortexed, kept on ice 30 min with continuous shaking, and centrifuged for 20 min at 4°C (4000 rpm). Supernatant was filtered through a 0.45 μm filter into a new Falcon tube and 1 mL was aliquoted and stored at -20°C as a pre-experiment input sample. We generated 2 mL aliquots from the remaining samples and added 5 μg of antibodies per gram of tissue. 20 μL of prepared Dynabeads (Life technologies) magnetic beads (ie, washed 3 times in wash buffer (20 mM Tris HCL pH 7.5, 5 mM MgCl_2_, 300 mM NaCl, 0.1% NP-40, 1% protease inhibitor, 100 units/mL RNase OUT), were added to each sample. After 2 h incubation on a rotation wheel at 4°C, the beads were washed 3 times and resuspended in 500 μL of washing buffer. The washing buffer was then discarded and replaced by 250 μL of elution buffer prepared according to [44] (100 mM NaHCO3, 1% SDS, 100 units/mL RNase OUT (Invitrogen) in 0.1% DEPC water), and the tubes were incubated 15 min at 65°C with agitation. Supernatant was transferred to fresh tubes and elution was repeated once. The two eluates were finally combined. Samples were treated with 0.08 μg/μL proteinase K for 15 min at 50°C. RNA was extracted following the recommendations from Applied Biosystems for TRI Reagent^®^ Solution, starting by adding 1.2 mL of TRI Reagent to the samples. Stem loop PCR small RNAs extracted from RNA-IPs were treated with DNase to remove potential DNA contamination using the TURBO DNA-free kit (AM1907, Ambion Life Technologies). DNA-free samples, 50 μM of stem-loop primers (listed in S2 Table), 10 mM of dNTP and nuclease-free water were mixed to reach a final volume of 13 μL. Stem-loop reverse transcription was performed following the recommendations from [45] and the resulting double-stranded cDNAs were used for PCR. 1 μL of cDNA was mixed with Red Taq 2x (Promega), and 0,25 μM of universal reverse primer (complementary to the stem loop one) and a specific forward primer designed to match the *b1TR* siRNAs. 20 μL reactions were denatured for 2 min at 94°C, and went through 40 cycles of 15 s at 94°C and 1 min at 60°C. Product visualization was performed by electrophoresis into 2% agarose gels (Lonza) in TBE 0.5X supplemented with 0.5 μg/mL BET for 40 min at 100 V. The Promega 100bp DNA Ladder Molecular Weight Marker was used. To verify cDNAs derived from *b1TR* siRNAs, amplified products were recovered from the gel using the QIAquick gel extraction kit (QIAGEN) and cloned in DH5α competent cells (Invitrogen) using the pGEM-T Easy Vector Systems protocol (Promega) and an LB-ampicillin selective medium. Colonies were genotyped using the T7/SP6 primers (Promega). Plasmids from selected colonies were isolated using the QIAprep Spin Miniprep Kit (QIAGEN) and sequenced (Beckman Coulter Genomics, Inc., UK).

### Small RNA sequencing

Small RNAs extracted from RNA-IPs were migrated on a 1.5% agarose gel and recovered from the corresponding bands using the Monarch DNA Gel Extraction kit (NEB #T1020 New England Biolab). RNA samples were used to prepare libraries using the NEBNext Multiplex Small RNA Library Prep Set (NEB #E7300S New England Biolab). The final PCR enrichment was performed using 15 cycles. Samples were quantified with Qubit and Agilent Bioanalyzer using the DNA high-sensitivity assays and sequenced on a NextSeq550 machine at the CSHL Genome Center.

### Small RNA seq analysis

Raw reads were cleaned up using Trimmomatic (Version 0.38) with the following parameters 2:30:5 LEADING:3 TRAILING:3 SLIDINGWINDOW:4:15 MINLEN:15 MAXLEN:35. Cleaned reads were first aligned (up to two mismatches allowed) to the maize reference genome B73 version 5 using Bowtie 1 (Version 1.2.2) with the—best -k 2 options for multiple mapping (only two valid alignment are reported and the best one is reported). Mapped reads coverage into 0.5 Mb genome windows was generated using the coverage utility of the Bedtools suite [46]. For a better resolution, reads were also aligned (up to two mismatches allowed) to the *b1TRs* and their 100-kb flanks using Bowtie 1 (Version 1.2.2) with -m 7—strata—best options for multiple mapping (reads with more than 7 alignments reported were discarded). They were intersected into 50-bp genome windows using Bedtools coverage.

### Quantification of plant pigmentation

Adapting a protocol from [47], we collected and froze stem tissue from the seventh leaf of plants at 56 days post-seeding (dps) with light stem pigmentation (heterozygous *mop1-1*), intermediate stem pigmentation (plants from cross 2) and dark purple stem pigmentation (homozygous *mop1-1*). 1 g of tissues was grinded in liquid nitrogen and incubated in 30 mL of methanol:water (70:30) for 24 h at 4°C. Tubes were then centrifugated at 5,000 g for 30 min, and the supernatant was collected and centrifugated for 10 more minutes. Supernatant was then assessed for absorbance at 550 nm. Differences in absorbance between the 3 phenotypes was tested using the Kruskal-Wallis test and a multiple pairwise comparison test. For plants at 46 dps, light, intermediate and dark purple stem pigmentation was estimated visually, using the recognizable green area around nodes (which is not observed in *mop1-1*).

## Supporting information

S1 Fig siRNA chromosome coverage on the B73 reference genome (version 5). siRNAs were extracted from AGO104 IPs in immature ears of three genetic backgrounds (B73, *Mm* and *mm*) with 2 technical and biological repeats. Reads were normalized in each sample using the TPM procedure. Colored highlights are the positions of the centromeres and the four known paramutation loci in maize (*p1* on chromosome 1, *b1* on chromosome 2, *pl1* on chromosome 6 and *r1* on chromosome 10).
(DOCX)

S2 Fig Full unedited digital image of the stem loop gel before cropping of irrelevant parts. Dotted boxes show the panels used in Fig 1b. Arrows indicate the expected 67-bp band.
(DOCX)

S1 Table AGO104 antibody characteristics. (DOCX)

S2 Table Primer sequences used for siRNA stem loop PCR. (DOCX)

S3 Table Primer sequences used for genotyping. The *Mutator* primer was associated with the Forward primers.
(DOCX)
