# Supplementary material for: AGO104 is a RdDM effector of paramutation at the maize b1 locus
Source: PLoS One. 2022 Aug 30;17(8):e0273695. doi: 10.1371/journal.pone.0273695 (PMC9426929; doi:10.1371/journal.pone.0273695)
Supplement: S1 Fig — siRNAs were extracted from AGO104 IPs in immature ears of three genetic backgrounds (B73, Mm and mm) with 2 technical and biological repeats. Reads were normalized in each sample using the TPM procedure. Colored highlights are the positions of the centromeres and the four known paramutation loci in maize (p1 on chromosome 1, b1 on chromosome 2, pl1 on chromosome 6 and r1 on chromosome 10). (DOCX) [file pone.0273695.s001.docx]

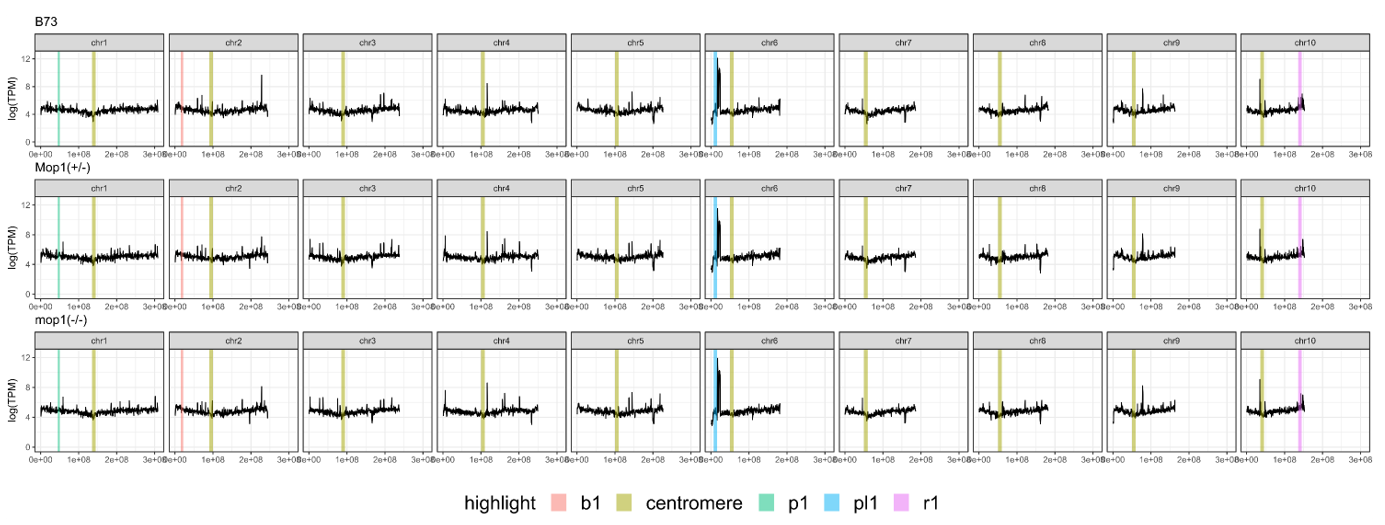


**Figure S1** siRNA chromosome coverage on the B73 reference genome (version 5).

siRNAs were extracted from AGO104 IPs in immature ears of three genetic backgrounds (B73, *Mm* and *mm*) with 2 technical and biological repeats. Reads were normalized in each sample using the TPM procedure. Colored highlights are the positions of the centromeres and the four known paramutation loci in maize (*p1* on chromosome 1, *b1* on chromosome 2, *pl1* on chromosome 6 and *r1* on chromosome 10).
