# Supplementary material for: AGO104 is a RdDM effector of paramutation at the maize b1 locus
Source: PLoS One. 2022 Aug 30;17(8):e0273695. doi: 10.1371/journal.pone.0273695 (PMC9426929; doi:10.1371/journal.pone.0273695)
Supplement: S2 Fig — Dotted boxes show the panels used in Fig 1b. Arrows indicate the expected 67-bp band. (DOCX) [file pone.0273695.s002.docx]

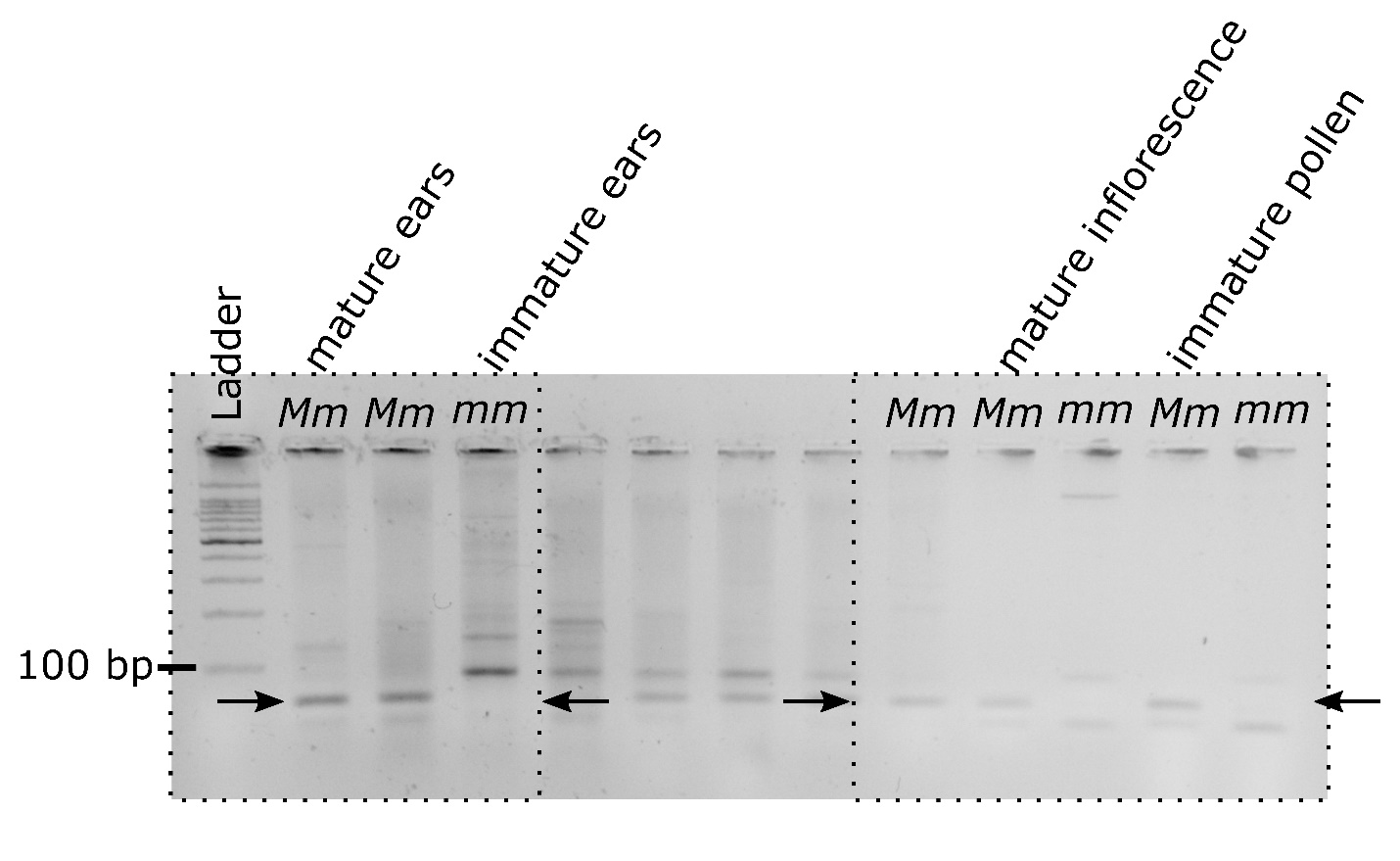
**Fig. S2** Full unedited digital image of the stem loop gel before cropping of irrelevant parts. Dotted boxes show the panels used in Fig 1b. Arrows indicate the expected 67-bp band.
