# Supplementary material for: AGO104 is a RdDM effector of paramutation at the maize b1 locus
Source: PLoS One. 2022 Aug 30;17(8):e0273695. doi: 10.1371/journal.pone.0273695 (PMC9426929; doi:10.1371/journal.pone.0273695)
Supplement: S1 Table — (DOCX) [file pone.0273695.s003.docx]

**Table S1** AGO104 antibody characteristics.

| Target protein | Target peptide | Peptide position | Organism |
| --- | --- | --- | --- |
| AGO104 | SERICKEQTFPLRQR | 326-341 | Rabbit |
