# Supplementary material for: AGO104 is a RdDM effector of paramutation at the maize b1 locus
Source: PLoS One. 2022 Aug 30;17(8):e0273695. doi: 10.1371/journal.pone.0273695 (PMC9426929; doi:10.1371/journal.pone.0273695)
Supplement: S2 Table — (DOCX) [file pone.0273695.s004.docx]

**Table S2** Primer sequences used for R3 siRNA stem loop PCR.

| siRNA sequence | Size (nt) | Stem loop primer | TM | Forward primer | TM |
| --- | --- | --- | --- | --- | --- |
| ATGATTTGTGGGTCCGATGGCATA | 24 | GTCGTATCCAGTGCAGGGTCCGAGGTATTCGCACTGGATACGATATGCC | 73 | TGCCGATGATTTGTGGGTCC | 59 |
