# Supplementary material for: AGO104 is a RdDM effector of paramutation at the maize b1 locus
Source: PLoS One. 2022 Aug 30;17(8):e0273695. doi: 10.1371/journal.pone.0273695 (PMC9426929; doi:10.1371/journal.pone.0273695)
Supplement: S3 Table — The Mutator primer was associated with the Forward primers. (DOCX) [file pone.0273695.s005.docx]

**Table S3** Primer sequences used for genotyping. The *Mutator* primer was associated with the Forward primers.

|  | Forward | Reverse | Mutator |
| --- | --- | --- | --- |
| *mop1-1* | TCTCCACCGCCCACTTGAT | ATGGCCAGCAGGGTGTCGCAGAT | AGAGAAGCCAACGCCAWCGCCTCYATTTCGTC |
| *ago104-5* | TGTCTCCTGTATCAACGGGGTGGTC | CTATACCAGGCCTGTCAATCAGTAATCTC |  |
